# Deep Learning- and Transfer Learning-Based Super Resolution Reconstruction from Single Medical Image

**DOI:** 10.1155/2017/5859727

**Published:** 2017-07-06

**Authors:** YiNan Zhang, MingQiang An

**Affiliations:** ^1^School of Computer Science and Technology, Beijing Institute of Technology, Beijing 100081, China; ^2^College of Computer Science and Information Engineering, Tianjin University of Science and Technology, Tianjin 300222, China; ^3^College of Science, Tianjin University of Science and Technology, Tianjin 300222, China

## Abstract

Medical images play an important role in medical diagnosis and research. In this paper, a transfer learning- and deep learning-based super resolution reconstruction method is introduced. The proposed method contains one bicubic interpolation template layer and two convolutional layers. The bicubic interpolation template layer is prefixed by mathematics deduction, and two convolutional layers learn from training samples. For saving training medical images, a SIFT feature-based transfer learning method is proposed. Not only can medical images be used to train the proposed method, but also other types of images can be added into training dataset selectively. In empirical experiments, results of eight distinctive medical images show improvement of image quality and time reduction. Further, the proposed method also produces slightly sharper edges than other deep learning approaches in less time and it is projected that the hybrid architecture of prefixed template layer and unfixed hidden layers has potentials in other applications.

## 1. Introduction

Medical imaging [1] is an important tool to determine the presence of many diseases and analysis of experimental results. Enlarging medical images [2] can provide medical experts with more details for elevating diagnosis accuracy [3] in pathology research [4]. Therefore, the medical image enhancement becomes to be a hotspot. Further, enlarged medical images may substantially help computer-aided automatic detection [5]. For example, majority of single-detector spiral computer tomography (CT) [6] scanners and magnetic resonance imaging (MRI) [7] produce medical images as effective noninvasive examinations. Because of technical restrictions, such types of medical images are obtained in relatively low resolution (LR) and not suitable for further analyzing. Therefore, super resolution reconstruction (SRR) [8] methods can be used for solving this type of problems.

For medical images, many SRR methods were proposed. Those methods fuse some LR images from the same scene to one high-resolution (HR) image. Many corresponding machine learning [9] methods were proved effectively. Wang et al. [10] proposed a sparse representation-based SRR method in 2014. Rousseau et al. [11] proposed a SRR approach from multiple low-resolution images. In 2011, Liyakathunisa [12] presented a SRR method which uses progressively DCT- and zonal filter-based denoising. Those researches were proved effective and efficient; however, those methods are limited by two conditions: (1) the input images must be a set of LR images and (2) the training dataset must be big enough which contains LR and HR medical images in pairs.

Those two conditions raise the following problems in reality: (1) a set of low-resolution medical images cannot be obtained for potential reasons, such as costs to patients, and (2) to machine learning approach, a big training dataset of LR and HR medical images is a giant cost in commercial project. For solving those problems, three conventional methods have been widely used. Nearest neighbor interpolation, bilinear interpolation, and cubic convolution interpolation are widely used [13, 14]. However, those three reconstruction approaches produce high-resolution image with blur edges or image aliasing [15]. In medical field, a better SRR method is needed in urgent. Combining deep learning with transfer learning to reconstruct a HR image from one single LR image becomes to be a feasible and practical solution.

In this paper, we adapt deep learning [16] and transfer learning [17] to achieve SRR for medical images. First, a deep conventional neural network (DCNN) is designed for fulfilling SRR. Second, SIFT feature-based transfer learning is used to enlarge the training dataset of the DCNN by using the public image dataset. Finally, the trained DCNN can reconstruct a HR medical image from a given LR medical image. The major contributions of this paper are the following:

1. Using deep learning to achieve better SRR result than conventional methods
2. Using transfer learning to enlarge training dataset for DCNN
3. Transfer learning can reduce costs of preparing medical images in reality
4. SIFT feature-based transfer learning and DCNN can offer sharper edge
5. Proposing a hybrid DCNN structure which contains a prefixed template layer.

The rest of this paper is organized as follows: Section 2 shows SRR in medical research and related works, Section 3 presents the proposed method, and Section 4 shows experiments and results. In Section 5, a conclusion is drawn.

## 2. Backgrounds and Related Works

**Figure 1:**
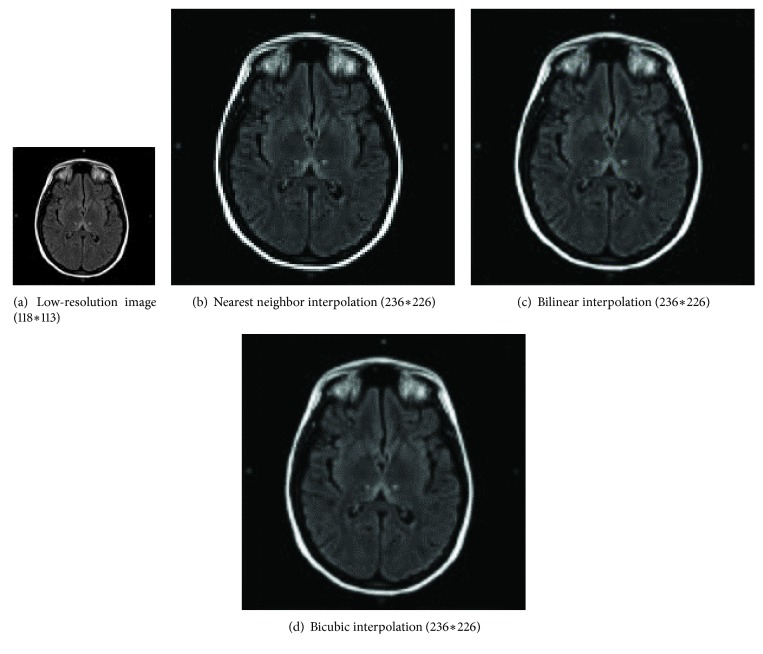
Three conventional SRR methods (MRI: brain).

There are three conventional super resolution reconstruction methods: nearest neighbor (NN) interpolation [18], bilinear interpolation [19], and bicubic interpolation [20]. An example of SRR for medical image (MRI: brain) is shown in Figure 1.

Figure 1(a) is a LR. Figures 1(b), 1(c), and 1(d) are results of those three conventional SRR methods. Advantages and disadvantages of those conventional SRR methods can be summarized in Table 1.

Plenge et al. [21] proposed a SRR method by using cross-scale self-similarity in multislice MRI. Tao et al. [22] showed an SSR method which is SRR of late gadolinium-enhanced MRI from multiple views in 2014. And Zhao et al. [23] proposed a multiframe SRR algorithm based on diffusion tensor regularization term in 2014.

Many effective SRR methods are proposed, but most of them are based on a group of LR medical images. Furthermore, nearest neighbor interpolation, bilinear interpolation, and bicubic interpolation can achieve SRR result for single medical image task now, but a better SRR method is needed in medical research and clinical diagnosis.

## 3. The Proposed Method

**Figure 2:**
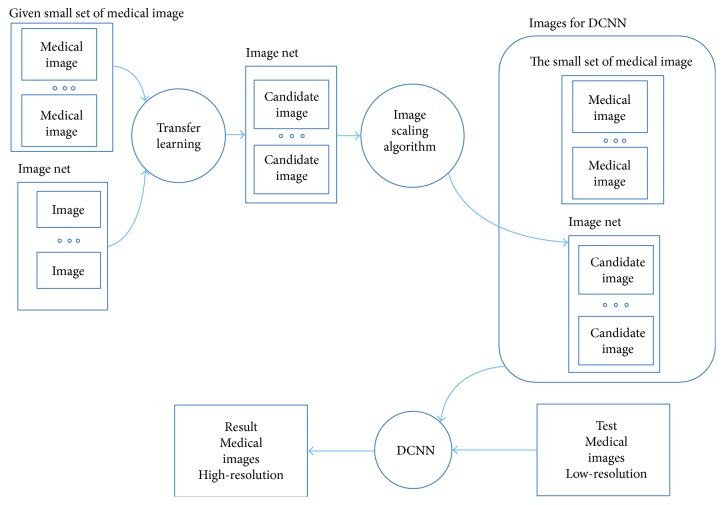
Overview: deep learning- and transfer learning-based SRR for single medical image.

The proposed method includes three distinctive parts of algorithms/techniques: (1) SIFT feature-based transfer learning, (2) image scaling-down algorithm, and (3) deep learning (deep convolutional neural network (DCNN)). Image scaling-down algorithm is a conventional algorithm. Using SIFT feature-based transfer learning and a hybrid DCNN structure is the major contribution in this paper. As Figure 2 shows, a small set of medical image samples is offered first. After the processing of SIFT feature-based transfer learning and image scaling-down algorithm, a big set of training dataset is prepared to train deep neural network. Finally, one LR image can be reconstructed to one HR image. Details are shown in Sections 3.1.1–3.1.5. Because this work is an improvement of SRCNN which was proposed by Dong et al. [24], Sections 3.1.2–3.1.5 have likely expressions as in reference [24].

### 3.1. Hybrid Deep Convolutional Neural Network Architectures

**Figure 3:**
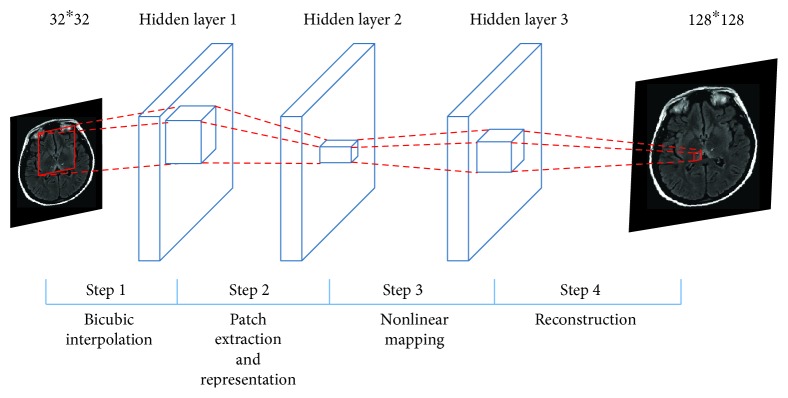
Deep convolutional neural network for medical image SRR.

We improve the SRCNN [24] structure and present a hybrid structure of DCNN in this section. The proposed DCNN contains three layers; it has one prefixed bicubic interpretation layer by mathematics deduction and two convolutional layers which learn from given training images. As Figure 3 shows, the LR image is the input and the HR image is the output. Three hidden layers are designed for different tasks. The hidden layer 1 is a bicubic interpretation template layer which is used to fulfill bicubic interpolation. The hidden layer 2 is designed for patch extraction and representation, and the hidden layer 3 is designed for nonlinear mapping. Finally, a reconstruction result is produced at the end of DCNN. The size of hidden layer 1 is *f*_1_^∗^*f*_1_^∗^*n*_1_, the size of hidden layer 2 is *f*_2_^∗^*f*_2_^∗^*n*_2_, and the size of hidden layer 3 is *f*_3_^∗^*f*_3_^∗^*n*_3_.

Steps of Figure 3 are as follows:

*Step 1*. This step fulfills the fast-bicubic interpolation; details of building this bicubic interpretation template layer are shown in Section 3.1.1.

*Step 2*. In this step, a convolutional layer achieves patch extraction and representation; details are shown in Section 3.1.2.

*Step 3*. This step maps high-dimensional vectors into another high-dimensional space; details are shown in Section 3.1.3.

*Step 4*. This reconstruction step produces the final HR image; details are shown in Section 3.1.4.

#### 3.1.1. Step 1: Prefixed Bicubic Interpolation Layer

**Figure 4:**
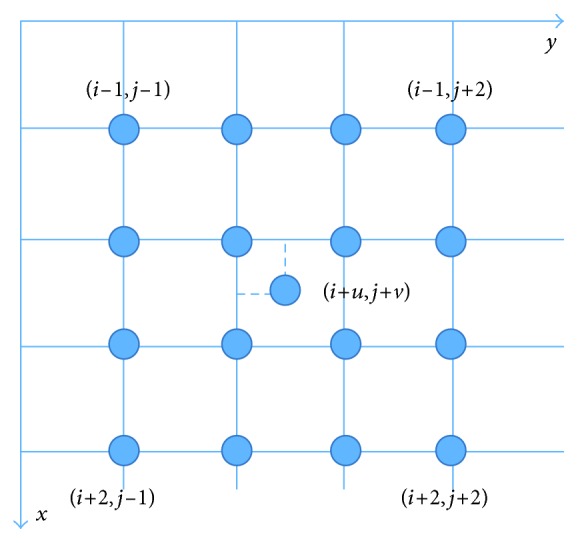
Bicubic interpretation.

Bicubic interpolation methods use extra points to fit the sampling functions, a critical problem is time cost. As Figure 4 shows, bicubic interpretation method uses 16 adjacent pixels as inputs and cubic polynomial approximation function of the best interpretation function sin(*πω*)/(*πω*) in theory.

*S*(*ω*) can be expressed as

(1) $$\begin{array}{c} S\left(\omega \right)=\left\{\begin{array}{l} 1-2{\left|\omega \right|}^{2}+{\left|\omega \right|}^{3},0\leq \left|\omega \right|<1\\ 4-8\left|\omega \right|+5{\left|\omega \right|}^{2}-{\left|\omega \right|}^{3},1\leq \left|\omega \right|<2\\ 0,\left|\omega \right|\geq 2. \end{array}\right. \end{array}$$

The pixel (*i* + *u*, *j* + *v*) can be calculated by

(2) $$\begin{array}{c} f\left(i+u,j+v\right)=\left[A\right]\times \left[B\right]\times \left[C\right], \end{array}$$

where

(3) $$\begin{array}{c} \left[A\right]=\left[S\left(1+v\right)\;S\left(v\right)\;S\left(1-v\right)\;S\left(2-v\right)\right], \end{array}$$

(4) $$\begin{array}{c} \left[B\right]=\left[\begin{array}{llll} f_{i-1,j-1} & f_{i-1,j} & f_{i-1,j+1} & f_{i-1,j+2}\\ f_{i,j-1} & f_{i,j} & f_{i,j+1} & f_{i,j+2}\\ f_{i+1,j-1} & f_{i+1,j} & f_{i+1,j+1} & f_{i+1,j+2}\\ f_{i+2,j-1} & f_{i+2,j} & f_{i+2,j+1} & f_{i+2,j+2} \end{array}\right], \end{array}$$

(5) $$\begin{array}{c} \left[C\right]=\left[\begin{array}{c} S\left(1+u\right)\\ S\left(u\right)\\ S\left(1-u\right)\\ S\left(2-u\right) \end{array}\right]. \end{array}$$

Then, the bicubic interpretation templates can be deduced as follows:

(6) $$\begin{array}{c} f\left(i+u,j+v\right)=\left[A\right]\times \left[B\right]\times \left[C\right]=\left[S\left(1+v\right)\;S\left(v\right)\;S\left(1-v\right)\;S\left(2-v\right)\right]\cdot \left[\begin{array}{llll} f_{i-1,j-1} & f_{i-1,j} & f_{i-1,j+1} & f_{i-1,j+2}\\ f_{i,j-1} & f_{i,j} & f_{i,j+1} & f_{i,j+2}\\ f_{i+1,j-1} & f_{i+1,j} & f_{i+1,j+1} & f_{i+1,j+2}\\ f_{i+2,j-1} & f_{i+2,j} & f_{i+2,j+1} & f_{i+2,j+2} \end{array}\right]\left[\begin{array}{c} S\left(1+u\right)\\ S\left(u\right)\\ S\left(1-u\right)\\ S\left(2-u\right) \end{array}\right]=f_{i-1,j-1}S\left(1+u\right)S\left(1+v\right)+f_{i-1,j}S\left(u\right)S\left(1+v\right)+f_{i-1,j+1}S\left(1-u\right)S\left(1+v\right)+f_{i-1,j+2}S\left(2-u\right)S\left(1+v\right)+f_{i,j-1}S\left(1+u\right)S\left(v\right)+f_{i,j}S\left(u\right)S\left(v\right)+f_{i,j+1}S\left(1-u\right)S\left(v\right)+f_{i,j+2}S\left(2-u\right)S\left(v\right)+f_{i+1,j-1}S\left(1+u\right)S\left(1-v\right)+f_{i+1,j}S\left(u\right)S\left(1-v\right)+f_{i+1,j+1}S\left(1-u\right)S\left(1-v\right)+f_{i+1,j+2}S\left(2-u\right)S\left(1-v\right)+f_{i+2,j-1}S\left(1+u\right)S\left(2-v\right)+f_{i+2,j}S\left(u\right)S\left(2-v\right)+f_{i+2,j+1}S\left(1-u\right)S\left(2-v\right)+f_{i+2,j+2}S\left(2-u\right)S\left(2-v\right)={\left[B\right]}^{*}T, \end{array}$$

where ^∗^ is the convolutional operation and

(7) $$\begin{array}{c} T=\left[\begin{array}{llll} S\left(1+u\right)S\left(1+v\right) & S\left(u\right)S\left(1+v\right) & S\left(1-u\right)S\left(1+v\right) & S\left(2-u\right)S\left(1+v\right)\\ S\left(1+u\right)S\left(v\right) & S\left(u\right)S\left(v\right) & S\left(1-u\right)S\left(v\right) & S\left(2-u\right)S\left(v\right)\\ S\left(1+u\right)S\left(1-v\right) & S\left(u\right)S\left(1-v\right) & S\left(1-u\right)S\left(1-v\right) & S\left(2-u\right)S\left(1-v\right)\\ S\left(1+u\right)S\left(2-v\right) & S\left(u\right)S\left(2-v\right) & S\left(1-u\right)S\left(2-v\right) & S\left(2-u\right)S\left(2-v\right) \end{array}\right]. \end{array}$$

The *u* and *v* (*u* ∈ [0, 1], *v* ∈ [0, 1]) can be discretized as follows.

If *u* ∈ [0, 1/4], *v* ∈ [0, 1/4], *u* and *v* can be set as its mean values; therefore, *u* = 1/8 and *v* = 1/8. From (1), (2), (3), (4), (5), (6), and (7), it can be deduced that

(8) $$\begin{array}{c} T_{1}=\frac{1}{2^{18}}\left[\begin{array}{llll} 2401 & -24353 & -3479 & 343\\ -24353 & 247009 & 35287 & -3479\\ -3479 & 35287 & 5041 & -497\\ 343 & -3479 & -497 & 49 \end{array}\right]. \end{array}$$

Therefore, *f*(*i* + *u*, *j* + *v*) = [*B*][*T*_1_]. Similarly, it can deduce 16 templates in total.  Table 2 shows all 16 conditions and corresponding discretized parameters.

Table 3 are the solutions of those 16 templates in Table 2. The hidden layer 1 can be built by those 16 templates, and those precalculated templates are weights of neurons. In the DCNN training step, this layer is constant and the weights of hidden layers 2-3 need to be updated in the following steps.

**Figure 5:**
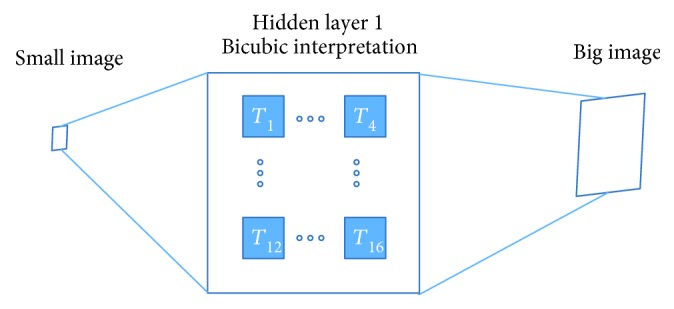
Hidden layer 1: fast bicubic interpretation.

Therefore, 16 templates in hidden layer 1 can fulfill the bicubic interpretation method as Figure 5 shows. Further, between the input and output images, there is only a multiple layer neural networks rather than a combination of bicubic interpretation and neural networks [24].

#### 3.1.2. Step 2: Patch Extraction and Representation

This step extracts patch from the results of fast bicubic interpolation layer and maps patch into high-dimensional space as vectors. The dimensionality of these vectors equals a set of feature mapping.

**Figure 6:**
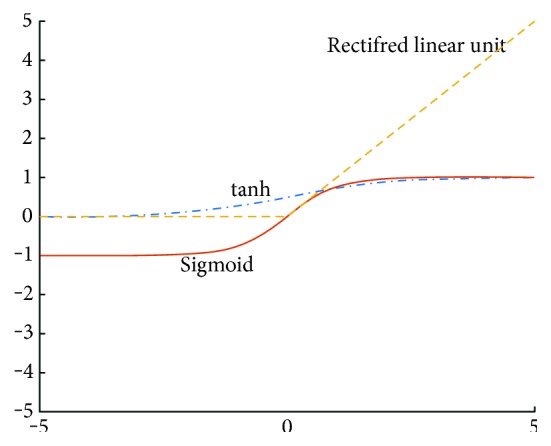
Plot of ReLU and classic activation function.

Hidden layer 2 contains a group of rectified linear unit (ReLU, max (0, *x*)) [25]. Classic activation function and the ReLU are plotted in Figure 6. To simplify, this layer can be expressed like an equation of a single ReLU unit. Therefore, *F*_2_ can be defined as follows:

(9) $$\begin{array}{c} F_{2}\left(Y\right)=\max\left(0,W_{2}Y+B_{2}\right), \end{array}$$

where *W*_2_ is the weight of neurons of hidden layer 2, *B*_2_ is biases, *W*_2_ is of a size *f*_2_^∗^*f*_2_^∗^*n*_2_, *f*_2_ is the spatial size of a filter, *n*_2_ is the number of filters, and *B*_2_ is an *n*_2_-dimensional vector. Equation (9) implies that a group of *f*_2_^∗^*f*_2_^∗^*n*_2_ ReLU neurons can be denoted as a single ReLU for simplicity.

#### 3.1.3. Step 3: Nonlinear Mapping

This step fulfills nonlinear mapping. The vectors are mapped in another high-dimensional space for patch extraction and representation layer. This mapping is for representing another set of features.

Like layer 2, the operation of the hidden layer 3 is

(10) $$\begin{array}{c} F_{3}\left(Y\right)=\max\left(0,W_{3}Y+B_{3}\right). \end{array}$$

Here, *W*_3_ is the weight and it is of a size *n*_1_^∗^1^∗^1^∗^*n*_3_ and *B*_3_ is *n_3_*-dimensional.

#### 3.1.4. Step 4: Reconstruction

This step generates the result of HR image by aggregating the above patch representations.

(11) $$\begin{array}{c} F_{4}\left(Y\right)=W_{4}F_{3}\left(Y\right)+B_{4}. \end{array}$$

Here, *W*_4_ is of a size *n*_2_^∗^*f*_3_^∗^*f*_3_ and *B*_4_ is an *n*_4_-dimensional vector. This step does not involve ReLU activation function.

#### 3.1.5. Learning Procedure of the Proposed DCNN

In the learning procedure, the mapping function *F* requires the estimation of a set of parameters *θ* = {*W*_*i*_, *B*_*i*_}, *i* = 2, 3, 4. *W*_*i*_ and *B*_*i*_ are weights and biases of neurons that should be obtained as above steps mentioned, and *W*_1_ and *B*_1_ of hidden layer 1 are precalculated in Section 3.1.2. For minimizing the loss between ground-truth HR image and the reconstructed image, it can use mean squared error (MSE) to indicate the loss function:

(12) $$\begin{array}{c} L\left(\theta \right)=\frac{1}{n}\overset{n}{\underset{i=1}{\sum }}{\left\|F\left(Y_{i};\theta \right)-X_{i}\right\|}^{2}, \end{array}$$

where *n* is the number of training samples. The loss can be minimized by using stochastic gradient descent with back propagation algorithm.

### 3.2. SIFT Image Feature-Based Evaluation Strategy for Transfer Learning

Many machine learning approaches hypothesize that the training and test dataset are drawn from the same feature space and they are in the same distribution. Once the distribution changes, lots of modules have to be rebuilt. In real-world applications, to collect sufficient training data is expensive or impossible. Therefore, it would be nice if reducing the effort of collecting the training data and the transfer learning would be desirable.

**Figure 7:**
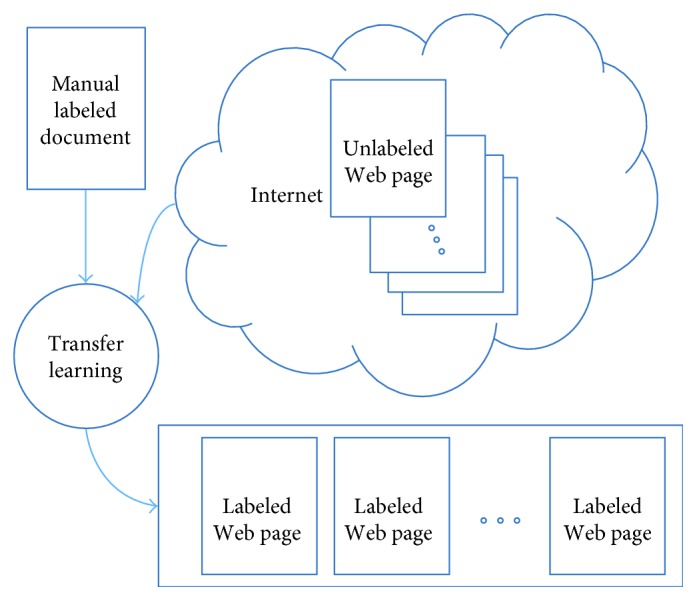
Instance-based transfer learning example: Web document classification.

One typical example is Web document classification; this is an instance-based transfer learning example. Once a document in the area of Web document classification is offered with manual labeling, it would be helpful if the classification knowledge could be transferred into new Web pages with that manual labeled document. As Figure 7 shows, transfer learning can help classifier to obtain bigger training dataset and which is similar to the manual labeled samples.

**Figure 8:**
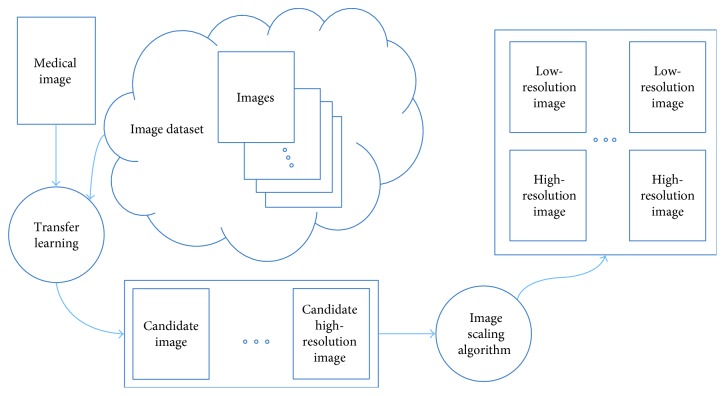
Transfer learning: SRR for medical images.

Inspired by the transfer learning as mentioned above, SRR for medical images can also employ transfer learning methodology. As Figure 7 shows, a big size of images can be obtained by using transfer learning, and then the DCNN can learn from sufficient medical images. However, input image and output image are in pairs and only one HR image can be easily obtained from public image dataset. With reversed thinking of SRR, we adapt image scaling-down algorithm to produce corresponding LR image; therefore, pair images are ready for DCNN to learn. As the assumption mentioned above “the training and test dataset are drawn from the same feature space and the same distribution,” we propose a SIFT image feature-based transfer learning as Figure 8 presents.

For obtaining sharper outlines, we use scale-invariant feature transform (SIFT) feature as the base which provides the distinctiveness, the robustness, and the generality. SIFT feature descriptor can capture structural properties robustly, and its points dominantly distribute among regions even color and texture change.

To a given image *I*(*x*,*y*), the Gaussian scale-space *L*(*x*, *y*, *D*) can be defined as conventional of scale variant Gaussian function *G*(*x*, *y*, *D*) and *I*(*x*,*y*), and they can be formulated as follows:

(13) $$\begin{array}{c} L\left(x,y,\sigma \right)=G\left(x,y,\sigma \right)\times I\left(x,y\right), \end{array}$$

(14) $$\begin{array}{c} G\left(x,y,\sigma \right)=\frac{1}{2\pi \sigma^{2}}e^{\left(x^{2}+y^{2}\right)/2\sigma^{2}}. \end{array}$$

In scale space, each pixel is compared to its surrounding 8 adjacent points and 18 neighboring points which are corresponding positions of two images adjacent scale of up and down in pyramid. If the pixel value is different to any of the 26 points, then this pixel is the candidate feature point.

It can be sampled in the neighborhood window and centered at the candidate feature points. Then, histogram is used to count the gradient direction of neighborhood pixels. The range of gradient histogram is from 0 to 360 degrees, and each column represents a direction in histogram.

Rotate the axis to the direction of the feature point which can ensure rotation invariance. Then, select an 8∗8 window centered at the feature point to calculate the gradient histogram of 8 directions in each small 4∗4 square. The accumulated values of each gradient direction are stored; therefore, a SIFT feature vector of 4∗4∗8 equal to128-dimensional vector is constituted.

We traverse all SIFT features in the given training image set. For each subregion of candidate images, we calculate the Euclidean distances to all the SIFT features, then sum up all of the distance, and define a mean of the sum as the distance among training images. It can be calculated as

(15) $$\begin{array}{c} D\left(S,T\right)=\frac{1}{m}\overset{m}{\underset{i=1}{\sum }}\left(\min_{1\leq j\leq n}\sqrt{\overset{t}{\underset{k=1}{\sum }}{\left(s_{ik}-t_{jk}\right)}^{2}}\right), \end{array}$$

where *m* is the number of SIFT features of image *S*, *t* = 128, *n* is the number of SIFT features of the training images, *s*_*ik*_ is the *k*_th_ element of the *i*_th_ feature vector of *S*, and *t*_*jk*_ is the *k*_th_ element of *j*_th_ feature vector of *T*.

*D*(*S*, *T*) denotes an average distance regarding all SIFT features among training images, and it is suitable for the case of detecting feature points from various images when applying instance-based transfer learning. In transfer learning set, images can be evaluated as follows:

(16) $$\begin{array}{c} \eta =\frac{N_{m}}{N_{t}}, \end{array}$$

where *N*_*m*_ and *N*_*t*_ indicate the number of matched SIFT feature pairs. Finally, the evaluation criterion is *η* ≥ *D*(*S*, *T*).

**Figure 9:**
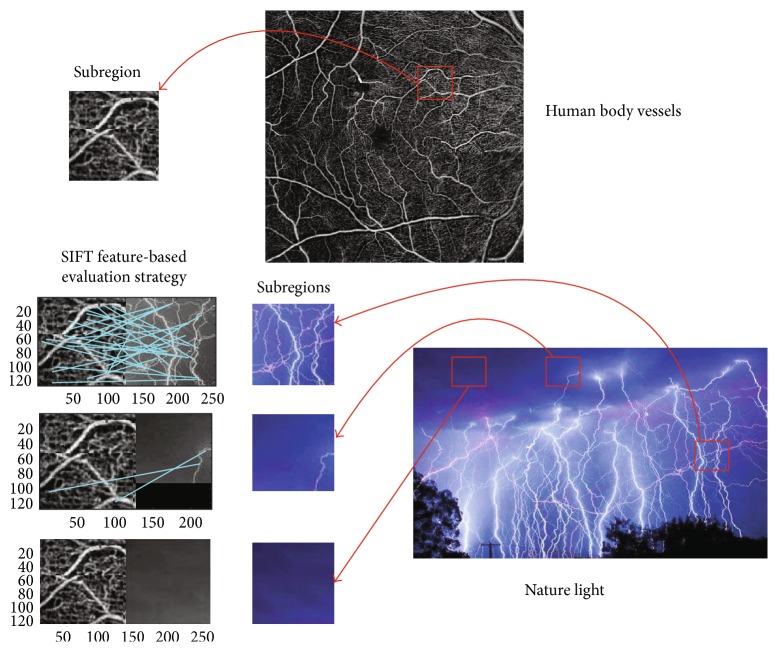
SIFT feature-based transfer learning example (vessels and light).

Figure 9 is an example for illustrating the proposed SIFT feature-based transfer learning. There are a medical image of angiography and a nature image of light. We cut three distinctive subregions of the image “light” and cut a subregion of vessels. We use SIFT feature to find matched points, and it can be found that the lights and vessels have lots of connections and those connections are key points in similar. If there is only one light, the connections reduce to 2 lines. However, if we compare the vessels and cloud part, there is no connected feature points. The proposed SIFT feature-based transfer learning intends to find a best match subregion and adds it into training set. Therefore, SIFT features can help to enlarge the training set for the DCNN.

## 4. Experiments and Results

### 4.1. Computation Environment and Training Set

Involved tools contain CUDA, Python language, and Open CV. The method selects candidate images from Image-Net dataset [26]. For comparison, the SRCNN [24] has been trained but without SIFT feature-based transfer learning. The training set for SRCNN is from the public medical image database [27].

**Figure 10:**
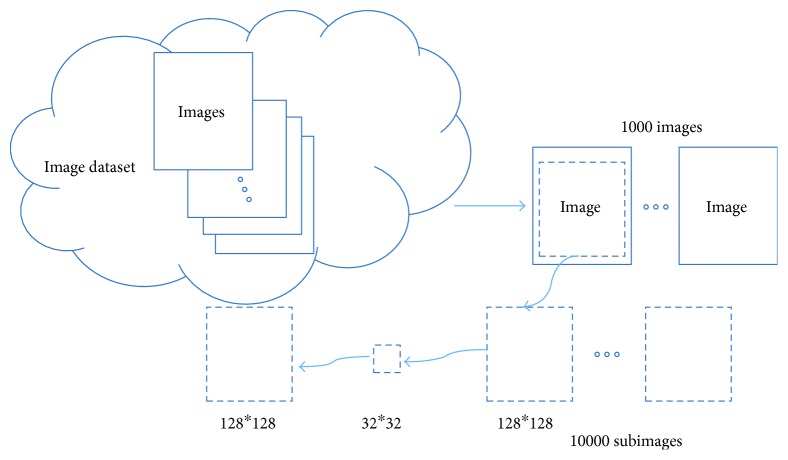
Preparation of training images.

As Figure 10 shows, a group of 1000 images are collected for the next step. Each training image is cut by the size of 128∗128 randomly and repeatedly. Finally, a set of 10000 images is done. The ground truth image which size is 128∗128 is scaled to 32∗32 as low-resolution image. Therefore, the 32∗32 images are used as low-resolution input image, and the ground truth images are used for training as the output of the deep convolutional neural network.

### 4.2. Quantitative Evaluation

We use peak signal-to-noise ratio (PSNR) for quantitatively image restoration quality. PSNR can be calculated by

(17) $$\begin{array}{c} PSNR=10\times \log_{10}\left(\frac{{\left(2^{n}-1\right)}^{2}}{MSE}\right), \end{array}$$

where MSE is the mean squared error

**Figure 11:**
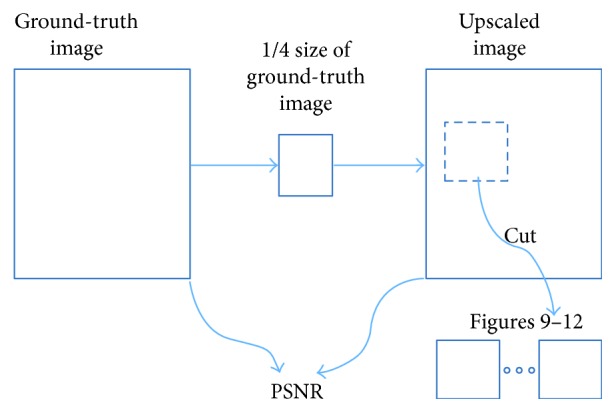
How to get PSNR and comparative figures.

For a fair comparison with conventional methods and SRCNN [24], we use medical images which were downloaded from research organization/authorities. Those four images can be downloaded freely by anyone. Brief information and the download weblink is listed in Table 2. We first scale those four images down to 1/4 size; therefore, PSNR can be calculated with results of SRR methods, as Figure 11 illustrates.

**Figure 12:**
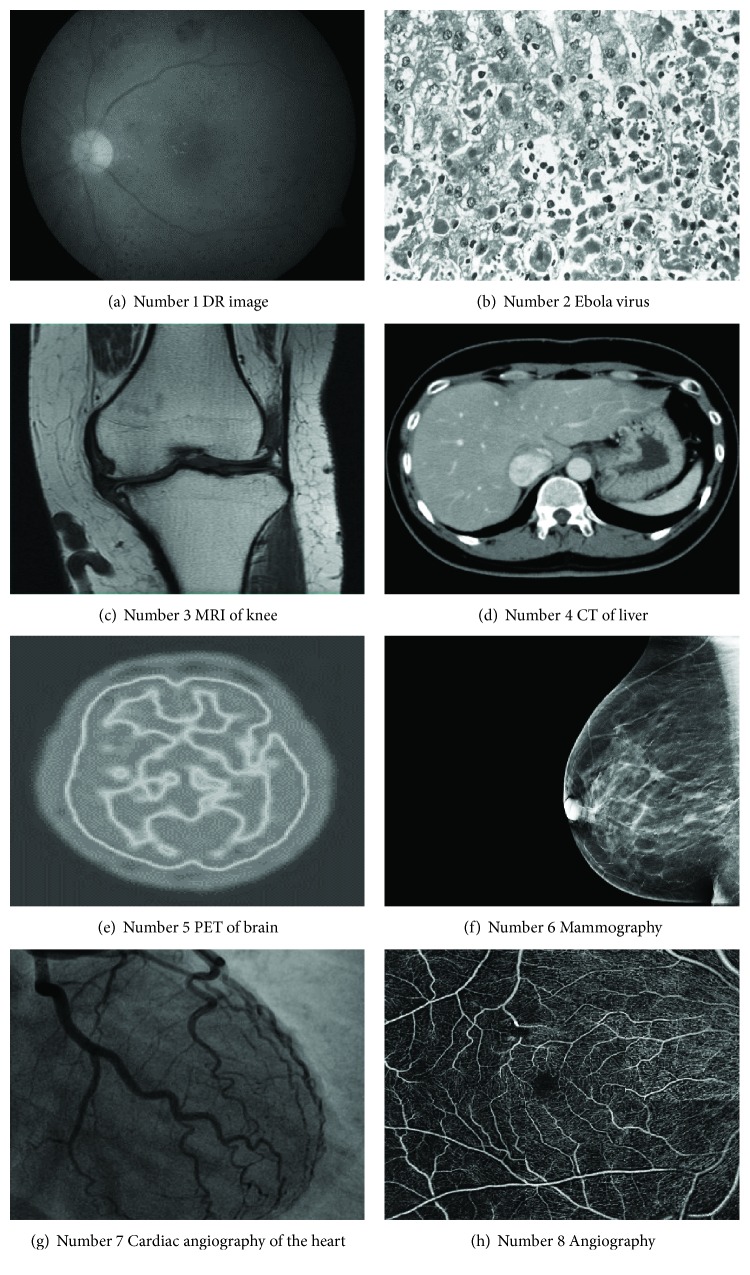
Ground-truth medical image.

**Figure 13:**
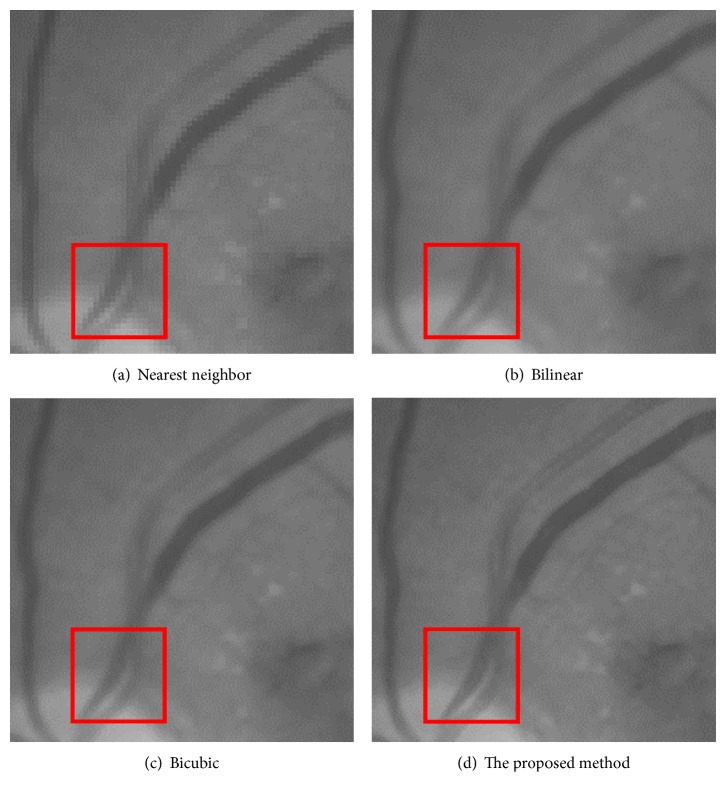
Diabetic retinopathy image.

**Figure 14:**
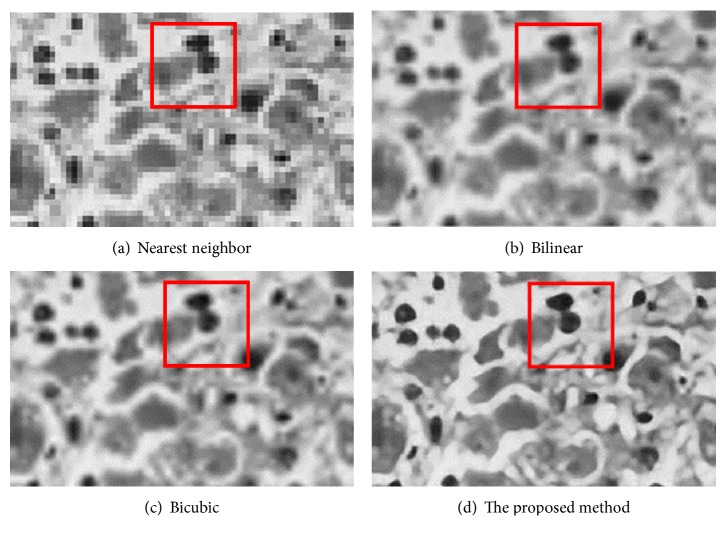
Ebola virus.

**Figure 15:**
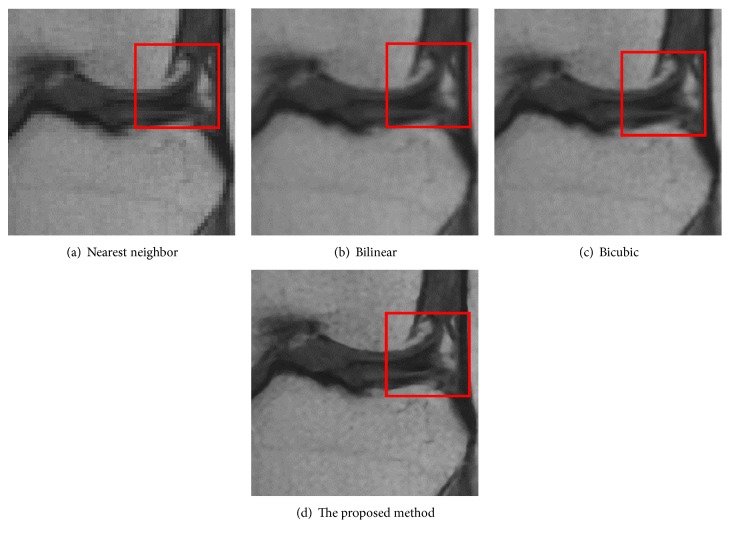
MRI knee.

**Figure 16:**
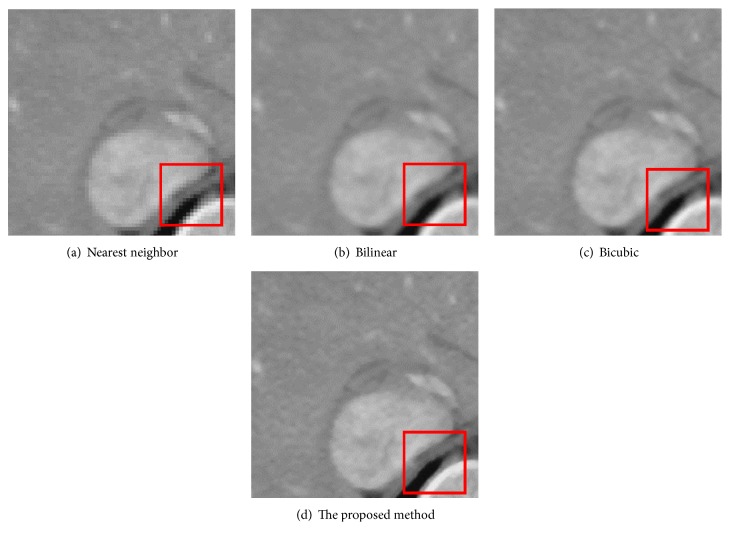
CT liver.

**Figure 17:**
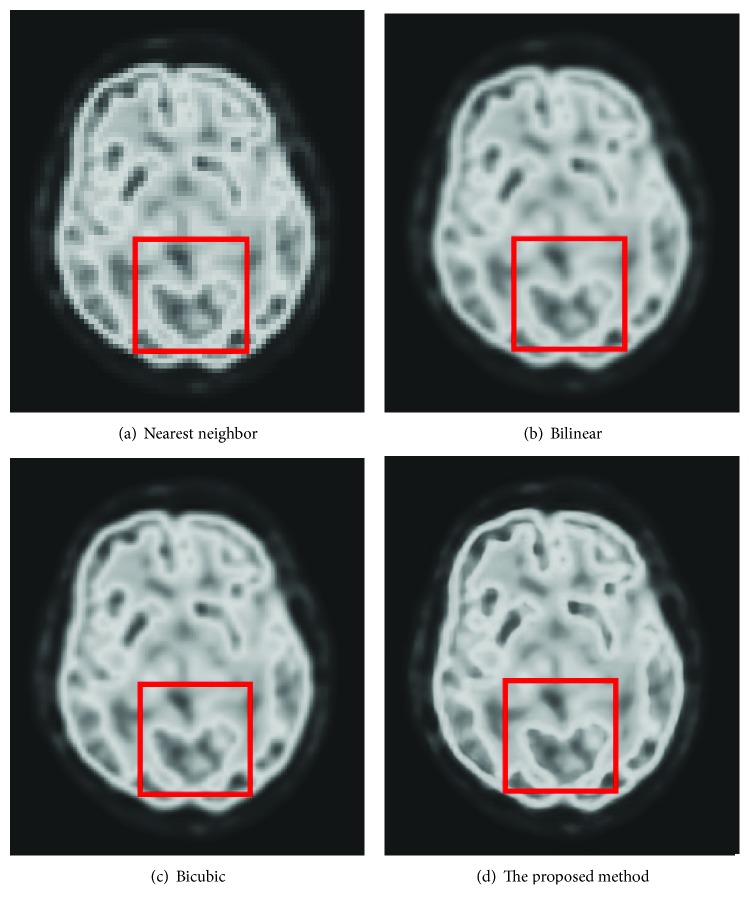
PET brain.

**Figure 18:**
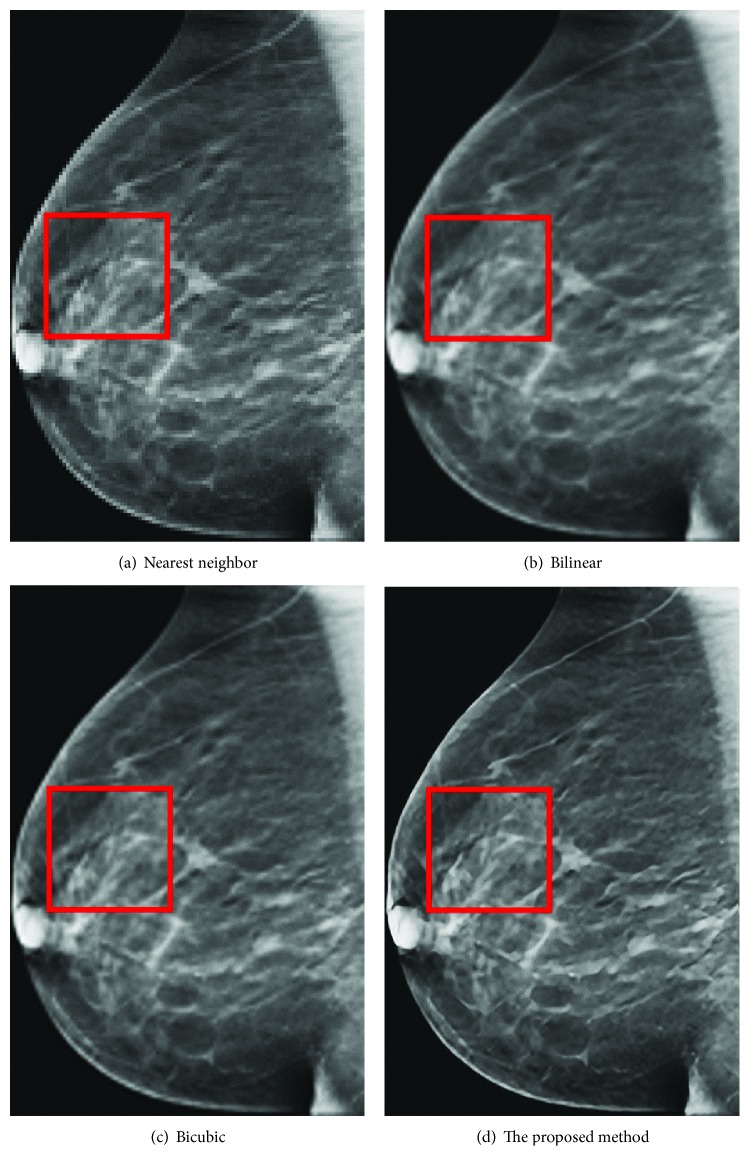
Mammography.

**Figure 19:**
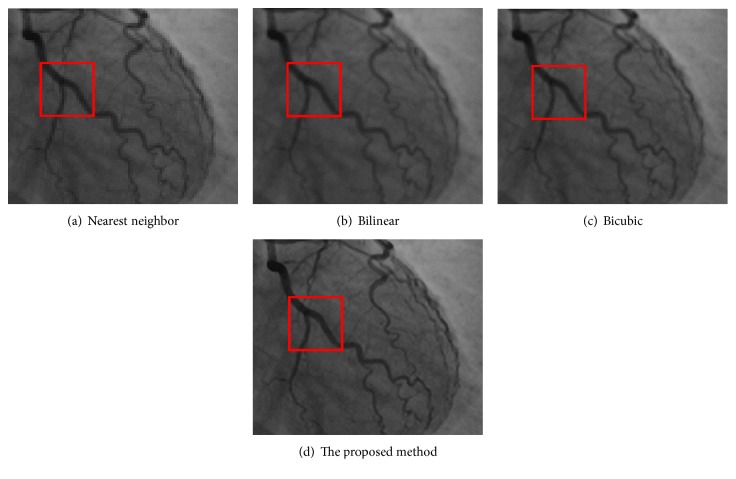
Cardiac angiography of the heart.

**Figure 20:**
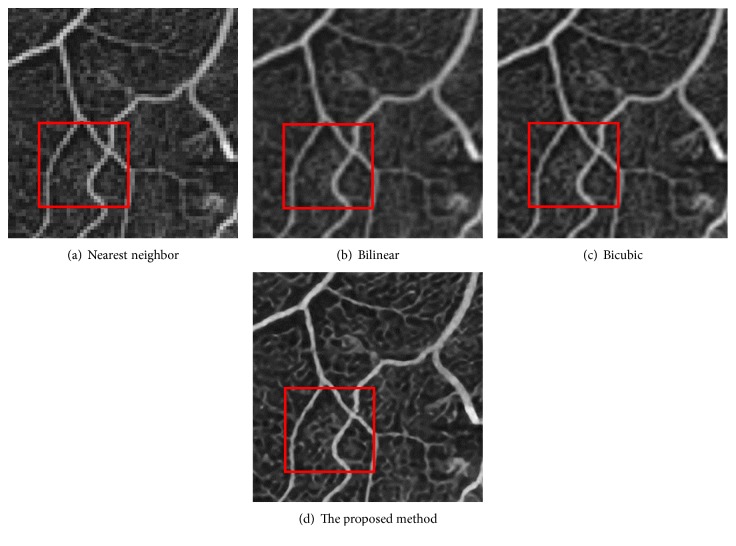
Angiography.

Figure 12 is the ground-truth images as Table 4 listed, and the resolution is the same as Table 2 presented. Figures 13, 14, 15, 16, 17, 18, 19, and 20 show the super resolution results of four different methods by upscaling factor 4. For intuitive comparison, each upscaled image is cut by a rectangle zone. As those images shown, the proposed method produces much shaper edges than other methods.

### 4.3. Experiment Results and Discussion

As an overview of Table 5, the proposed method gives the highest PSNR in 5 experiments. It is also can be seen from Figures 13–20 intuitively. Nearest neighbor interpolation gives the lowest PSNR for four images. Bilinear interpolation is slightly better than nearest neighbor interpolation. The bicubic interpolation is the best conventional method for four images. Completed with SRCNN, the proposed method achieves 0.04–0.07 higher in PSNR index. But, numbers 1, 3, and 4 experiments show that SRCNN still gives better PSNR than the proposed DCNN and the PSNR is between 0.03 and 0.05.

In Figure 3(), experimental results are presented. The image is about diabetic retinopathy research. Obviously, image aliasing exists in Figure 3(a) which is the result of nearest neighbor method. In Figure 3(b), the bilinear method gives more smooth edge of microvascular vessel (microblood vessel), but comparing Figure 3(b) with Figure 9()(c), the bicubic method gives a higher contrast method. In Figure 3()(d), the proposed method gives a sharper image and it should be noticed that the microvascular vessel and background had higher contrast than the other three methods.

In Figure 14, experimental results are about Ebola virus in microscope. Nearest neighbor method still gives an image with saw-tooth; this result cannot satisfy the needs of further analysis in medical research. Bicubic method shows better result than bilinear method, but the proposed method produces the best image among comparative methods.

SRR result of MRI image (knee) is shown in Figure 15. The proposed method shows a clearer meniscus and bone edges than the other methods. Bicubic method gives better results than nearest neighbor and bilinear method.

Figure 16 is the result of SRR for CT image (liver). Like Figures 13 and 14, the proposed method gives the best result. However, visual contrast among Figures 16(a), 16(b), 16(c), and 16(d) is not so obvious as Figures 14 and 15 show, the rectangle area of Figure 16(d) still presents a clear edge.

Figures 17–20 are results of PET brain, mammography, cardiac angiography, and angiography. Those medical images are processed by four methods, and subregions are cut for comparison. It can be found that the proposed DCNN can produce enlarged images with sharper edges in visual contract.

**Figure 21:**
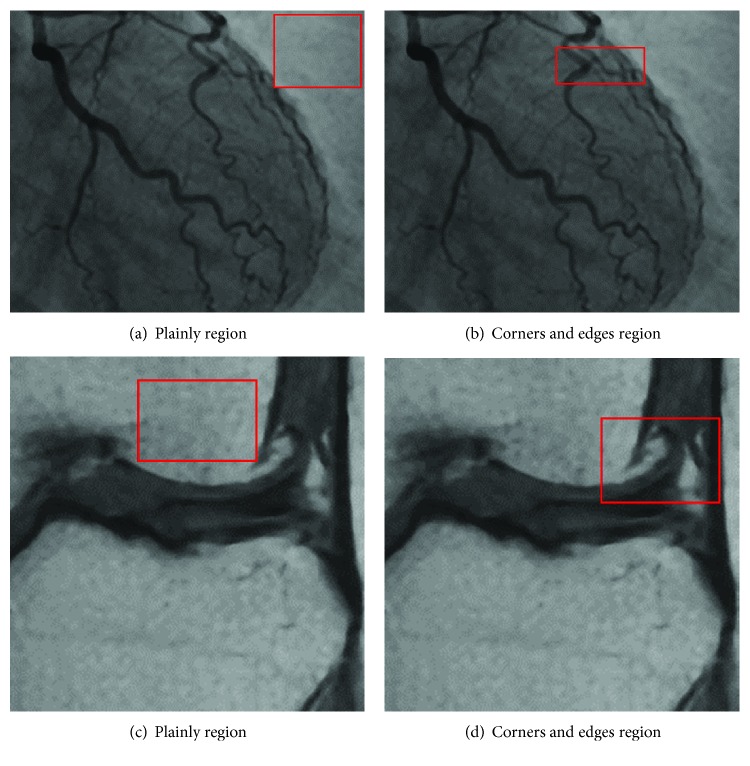
Comparison of different regions (PSNR).

Figure 21 shows comparison between the proposed method and SRCNN, and Table 6 presents the PSNR index of Figure 21. We choose two types of subregions which contain sharp edges or plainly texture, and we call them as “corners and edges region” and “plainly region.” By comparing the PSNR of those two types of regions, interesting details can be found. The SRCNN has bigger PSNR than the proposed DCNN in Figures 21(a) and 21(c); those two subregions are compared in plainly regions. It can be seen that the proposed DCNN is slightly higher than SRCNN in Figures 17(b) and 17(d) which are corner regions. As the previous example of Figure 9 illustrated in Section 3.2, the proposed DCNN learns from more subimages from the image database with the SIFT feature-based selection strategy.

(18) $$\begin{array}{c} f\left(i+u,j+v\right)=f_{i-1,j-1}S\left(1+u\right)S\left(1+v\right)+f_{i-1,j}S\left(u\right)S\left(1+v\right)+f_{i-1,j+1}S\left(1-u\right)S\left(1+v\right)+f_{i-1,j+2}S\left(2-u\right)S\left(1+v\right)+f_{i,j-1}S\left(1+u\right)S\left(v\right)+f_{i,j}S\left(u\right)S\left(v\right)+f_{i,j+1}S\left(1-u\right)S\left(v\right)+f_{i,j+2}S\left(2-u\right)S\left(v\right)+f_{i+1,j-1}S\left(1+u\right)S\left(1-v\right)+f_{i+1,j}S\left(u\right)S\left(1-v\right)+f_{i+1,j+1}S\left(1-u\right)S\left(1-v\right)+f_{i+1,j+2}S\left(2-u\right)S\left(1-v\right)+f_{i+2,j-1}S\left(1+u\right)S\left(2-v\right)+f_{i+2,j}S\left(u\right)S\left(2-v\right)+f_{i+2,j+1}S\left(1-u\right)S\left(2-v\right)+f_{i+2,j+2}S\left(2-u\right)S\left(2-v\right). \end{array}$$

Another comparison is running time. The proposed convolutional hidden layer 1 saves approximately half time costs than classic bicubic interpretation; therefore, it can save much time. As (18) shows, we can deduce the bicubic interpretation which has 28 floating-point multiplications and 41 floating-point additions. The proposed bicubic interpretation hidden layer needs 16 integer multiplications, 15 integer additions, 1 integer division, and 4.6 floating-point additions. Computational needs are shown in Table 7. Those values in Table 7 are derived from (1), (7), and (18) and Table 2. Therefore, the proposed DCNN is faster than SRCNN not only in practice but also in theory.

Table 8 shows the time costs of the eight distinctive medical images on a PC (Intel XEON E3-1230V3, 16G RAM). All images are preloaded in RAM, and then we processed those medical images by five methods and recorded the time costs. As Table 8 shows in milliseconds, NN is the fastest method and the SRCNN is slowest method. However, conventional methods such as NN, bicubic, and bilinear interpretation methods cannot meet the needs of medical image enlargement. Comparing SRCNN and the proposed method, the proposed method is slightly faster than SRCNN. It should be noticed that the training procedure processes lots of images; therefore, to save various time slices is meaningful for compute-intensive methods, such as deep learning.

The SRCNN is a novel super resolution reconstruction method, and we tried to improve three parts:

1. Prefixed template layer: A prefixed template layer saves costs by using mathematic deduction. On the other hand, training a convolutional layer with given training samples to fulfill bicubic interpretation is feasible. However, to train a convolutional layer requires various training images in pair. Therefore, we suggest to prefix the bicubic interpretation layer by mathematics deduction. Moreover, the proposed fixed templates may help other researchers and engineers to use them in real application easily, and they can deduce and verify those templates by their own.
2. Hybrid DCNN structure: Most researches focus on training deep neural networks (NN), and the whole NN is composed of unfixed parameters. The structure of our method combines fixed and unfixed parameters. Maybe, the combination of fixed and unfixed NN structure has undiscovered potentials in other applications.
3. Reducing costs and enhanced edge: The prefixed template layer can save more time than SRCNN, and the proposed SIFT feature-based transfer learning method guarantees the proposed DCNN can produce enlarged medical images with sharper edges.

To conclude, nearest neighbor method may be suitable for some occasions, but it definitely cannot satisfy SRR needs of medical images, such as microscope, CT, MRI, mammography, cardiac angiography, and angiography. Compared with the other conventional methods, bicubic gives better results than bilinear result; however, bicubic method still yields to the proposed method. The SRCNN is an effective and efficient DCNN architecture, but it lacks a faster convolutional interpretation layer. The bicubic interpretation hidden layer in the proposed method ensures a faster running speed than SRCNN, and the SIFT feature-based transfer learning provides sharper edges and corners region than SRCNN by selectively choosing training samples. Moreover, the bicubic interpretation hidden layer can provide an enlarged image which has continuous first and second derivative. This novel bicubic interpretation hidden layer has the potential to solve other image enhancement problems.

## 5. Conclusion

In this paper, a deep learning- and transfer learning-based super resolution reconstruction method has been presented. The proposed method aims to reconstruct a high-resolution image form one single low-resolution image. We propose a fast bicubic interpretation layer and SIFT feature-based transfer learning to speed up DCNN and to obtain sharper outlines; therefore, the proposed method can avoid collecting a great number of various medical images. Empirical experiments show that the proposed method can achieve better performance than other conventional methods. We suggest that this enhancement method is meaningful for clinical diagnosis, medical research, and automatic image analysis.

## Figures and Tables

**Table 1:** Advantages and disadvantages of three conventional SRR methods.

Interpolation method	Advantages	Disadvantages
Nearest neighbor	Easy to implement	Problem of image aliasing
	Very fast	

Bilinear	Antialiasing	Blur edges
	Considering with 4 nearest pixels	

Bicubic	Antialiasing	Slightly blur edges
	Considering with 16 nearest pixels	Relatively slow

**Table 2:** Bicubic interpretation template parameters.

Template	Conditions	Discretized parameters
*T*_1_	*v* ∈ [0, 1/4], *u* ∈ [0, 1/4]	Let *v* = 1/8 and *u* = 1/8
*T*_2_	*v* ∈ [0, 1/4], *u* ∈ [1/4, 2/4]	Let *v* = 1/8 and *u* = 3/8
*T*_3_	*v* ∈ [0, 1/4], *u* ∈ [2/4, 3/4]	Let *v* = 1/8 and *u* = 5/8
*T*_4_	*v* ∈ [0, 1/4], *u* ∈ [3/4, 1]	Let *v* = 1/8 and *u* = 7/8
*T*_5_	*v* ∈ [1/4, 2/4], *u* ∈ [0, 1/4]	Let *v* = 3/8 and *u* = 1/8
*T*_6_	*v* ∈ [1/4, 2/4], *u* ∈ [1/4, 2/4]	Let *v* = 3/8 and *u* = 3/8
*T*_7_	*v* ∈ [1/4, 2/4], *u* ∈ [2/4, 3/4]	Let *v* = 3/8 and *u* = 5/8
*T*_8_	*v* ∈ [1/4, 2/4], *u* ∈ [3/4, 1]	Let *v* = 3/8 and *u* = 7/8
*T*_9_	*v* ∈ [2/4, 3/4], *u* ∈ [0, 1/4]	Let *v* = 5/8 and *u* = 1/8
*T*_10_	*v* ∈ [2/4, 3/4], *u* ∈ [1/4, 2/4]	Let *v* = 5/8 and *u* = 3/8
*T*_11_	*v* ∈ [2/4, 3/4], *u* ∈ [2/4, 3/4]	Let *v* = 5/8 and *u* = 5/8
*T*_12_	*v* ∈ [2/4, 3/4], *u* ∈ [3/4, 1]	Let *v* = 5/8 and *u* = 7/8
*T*_13_	*v* ∈ [3/4, 1], *u* ∈ [0, 1/4]	Let *v* = 7/8 and *u* = 1/8
*T*_14_	*v* ∈ [3/4, 1], *u* ∈ [1/4, 2/4]	Let *v* = 7/8 and *u* = 3/8
*T*_15_	*v* ∈ [3/4, 1], *u* ∈ [2/4, 3/4]	Let *v* = 7/8 and *u* = 5/8
*T*_16_	*v* ∈ [3/4, 1], *u* ∈ [3/4, 1]	Let *v* = 7/8 and *u* = 7/8

**Table 3:** Bicubic interpretation template solutions.

$T_{1}=\frac{1}{2^{18}}\left[\begin{array}{rrrr} 2401 & -24353 & -3479 & 343\\ -24353 & 247009 & 35287 & -3479\\ -3479 & 35287 & 5041 & -497\\ 343 & -3479 & -497 & 49 \end{array}\right]$	$T_{2}=\frac{1}{2^{18}}\left[\begin{array}{rrrr} 3675 & -19355 & -11613 & 2205\\ -37275 & 196315 & 117789 & -22365\\ -5325 & 28045 & 16827 & -3195\\ 525 & -2765 & -1659 & 315 \end{array}\right]$

$T_{3}=\frac{1}{2^{18}}\left[\begin{array}{rrrr} 2205 & -11613 & -19355 & 3675\\ -22365 & 117789 & 196315 & -37275\\ -3195 & 16827 & 28045 & -5325\\ 315 & -1659 & -2765 & 525 \end{array}\right]$	$T_{4}=\frac{1}{2^{18}}\left[\begin{array}{rrrr} 343 & -3479 & -24353 & 2401\\ -3479 & 35287 & 247009 & -24353\\ -497 & 5041 & 35287 & -3479\\ 49 & -497 & -3479 & 343 \end{array}\right]$

$T_{5}=\frac{1}{2^{18}}\left[\begin{array}{rrrr} 3675 & -37275 & -5325 & 525\\ -19355 & 196315 & 28045 & -2765\\ -11613 & 117789 & 16827 & -1659\\ 2205 & -22365 & -3195 & 315 \end{array}\right]$	$T_{6}=\frac{1}{2^{18}}\left[\begin{array}{rrrr} 5625 & -29625 & -17775 & 3375\\ -29625 & 156025 & 93615 & -17775\\ -17775 & 93615 & 56169 & -10665\\ 3375 & -17775 & -10665 & 2025 \end{array}\right]$

$T_{7}=\frac{1}{2^{18}}\left[\begin{array}{rrrr} 3375 & -17775 & -29625 & 5625\\ -17775 & 93615 & 156025 & -29625\\ -10665 & 56169 & 93615 & -17775\\ 2205 & -10665 & -17775 & 3375 \end{array}\right]$	$T_{8}=\frac{1}{2^{18}}\left[\begin{array}{rrrr} 525 & -5325 & -37275 & 3675\\ -2\;765 & 28045 & 196315 & -19355\\ -1\;659 & 16827 & 117789 & -11613\\ 315 & -3195 & -22365 & 2205 \end{array}\right]$

$T_{9}=\frac{1}{2^{18}}\left[\begin{array}{rrrr} 2205 & -22365 & -3195 & 315\\ -11613 & 117789 & 16827 & -1659\\ -19355 & 196315 & 28045 & -2765\\ 3675 & -37275 & -5325 & 525 \end{array}\right]$	$T_{10}=\frac{1}{2^{18}}\left[\begin{array}{rrrr} 3375 & -17775 & -10665 & 2025\\ -17775 & 93615 & 56169 & -10665\\ -29625 & 156025 & 93615 & -17775\\ 5625 & -29625 & -17775 & 3375 \end{array}\right]$

$T_{11}=\frac{1}{2^{18}}\left[\begin{array}{rrrr} 2205 & -10665 & -17775 & 3375\\ -10665 & 56169 & 93615 & -17775\\ -17775 & 93615 & 156025 & -29625\\ 3375 & -17775 & -29625 & 5625 \end{array}\right]$	$T_{12}=\frac{1}{2^{18}}\left[\begin{array}{rrrr} 315 & -3195 & -22365 & 2205\\ -1659 & 16827 & 117789 & -11613\\ -2765 & 28045 & 196315 & -19355\\ 525 & -5325 & -37275 & 3675 \end{array}\right]$

$T_{13}=\frac{1}{2^{18}}\left[\begin{array}{rrrr} 343 & -3479 & -497 & 49\\ -3479 & 35287 & 5041 & -497\\ -24353 & 247009 & 35287 & -3479\\ 2401 & -24353 & -3479 & 343 \end{array}\right]$	$T_{14}=\frac{1}{2^{18}}\left[\begin{array}{rrrr} 525 & -2765 & -1659 & 315\\ -5325 & 28045 & 16827 & -3195\\ -37275 & 196315 & 117789 & -22365\\ 3675 & -19355 & -11613 & 2205 \end{array}\right]$

$T_{15}=\frac{1}{2^{18}}\left[\begin{array}{rrrr} 315 & -1659 & -2765 & 525\\ -3195 & 16827 & 28045 & -5325\\ -22365 & 117789 & 196315 & -37275\\ 2205 & -11613 & -19355 & 3675 \end{array}\right]$	$T_{16}=\frac{1}{2^{18}}\left[\begin{array}{rrrr} 49 & -497 & -3479 & 343\\ -497 & 5041 & 35287 & -3479\\ -3479 & 35287 & 247009 & -24353\\ 343 & -3479 & -24353 & 2401 \end{array}\right]$

**Table 4:** Public medical images for comparison.

Number	File name	Description	Provider	Download weblink
(1)	image008.png	Size: 1500∗1152 This image is from a public database of diabetic retinopathy detection	Lappeenranta University of Technology (LUT, in Finland)	http://www.it.lut.fi/project/imageret/diaretdb1/resources/images/ddb1_fundusimages/image008.png
(2)	7058_lores.jpg	Size: 700∗466 This micrograph of human liver tissue infected with the Ebola virus.	Centers for Disease Control and Prevention (CDC, in U.S.)	https://phil.cdc.gov/PHIL_Images/20050318/21149f4ebdee43fcaee990f40021450a/7058_lores.jpg
(3)	MRIofKnee.jpg	Size: 693∗779 MRI image of the knee	National Institute of Health (NIH, in U.S.)	https://www.nibib.nih.gov/sites/default/files/MRI%20of%20Knee.jpg
(4)	CaseKS11-CT-liverSOL-3.JPG	Size: 1114∗905 CT image of the liver	Department of Nuclear Medicine Biotracer Medicine, Kanazawa university (ac.jp, in Japan)	http://nucmed.w3.kanazawa-u.ac.jp/NMC/CaseKS11/CaseKS11-CT-liverSOL-3.JPG
(5)	300px-PET-image.jpg	Size: 300∗339 PET (positron emission tomography) image of the brain	http://wikimedia.org	https://upload.wikimedia.org/wikipedia/commons/thumb/c/c6/PET-image.jpg/300px-PET-image.jpg
(6)	ht_141204_senoclaire_3d_mammography_800 × 600.jpg	Size: 800∗600 Mammography	http://medscape.com	http://img.medscape.com/news/2014/ht_141204_senoclaire_3d_mammography_800x600.jpg
(7)	heart-385 × 330.jpg	Size: 385∗330 Cardiac angiography of the heart	http://ideviate.org	http://ideviate.org/wp-content/uploads/2012/05/heart-385x330.jpg
(8)	panoramic-cnv-octa.jpg	Size: 1360∗1346 Angiography	http://www.oct-optovue.com	http://www.oct-optovue.com/angioOCT-ARMD/oct-angiography-armd7_files/panoramic-cnv-octa.jpg

**Table 5:** The results of PSNR (dB).

Number and image name	Bicubic	The proposed method	Bilinear	NN	SRCNN
Number 1 DR	46.35	46.74	45.77	44.35	**46.79**
Number 2 Ebola	25.61	**27.29**	24.31	22.79	27.25
Number 3 MRI knee	36.67	37.74	35.33	33.04	**37.77**
Number 4 CT liver	39.27	**39.81**	36.96	32.49	27.74
Number 5 PET brain	37.71	37.21	34.61	30.11	**37.26**
Number 6 Mammography	30.64	**31.65**	30.13	29.49	31.60
Number 7 Cardiac angiography	36.28	**37.23**	35.56	34.47	37.19
Number 8 Angiography	25.96	**27.27**	24.98	24.13	27.21

**Table 6:** Comparison of edge and noncorner region in PSNR index (dB).

Image	Region	The proposed method	SRCNN
Number 4 MRI knee	Figure 21(d) Corners and edges region	**34.57**	34.12
	Figure 21(c) Plainly region	37.51	**37.65**

Number 7 Cardiac angiography	Figure 21(b) Corners and edges region	**36.22**	36.13
	Figure 21(a) Plainly region	42.79	**42.85**

**Table 7:** Running costs of bicubic interpretation and hidden layer 1 (integer and floating-point arithmetic).

Computational operation	Hidden layer 1	Bicubic interpretation
Integer addition	15 times	0 times
Integer division	1 time	0 times
Integer multiplications	16 times	0 times
Floating-point additions	4.6 times	41 times
Floating-point multiplications	0 times	28 times

**Table 8:** Overall comparisons of running time (in milliseconds).

Image	Methods				
	NN	Bicubic	Bilinear	SRCNN	The proposed method
Number 1 DR	8.075	14.790	14.655	63579.328	63565.847
Number 2 Ebola	8.000	10.518	11.613	11244.818	11234.735
Number 3 MRI knee	7.003	12.180	12.029	19231.110	19217.921
Number 4 CT liver	7.362	11.270	12.214	36315.960	36304.727
Number 5 PET brain	8.993	9.067	12.954	1986.092	1977.153
Number 6 Mammography	6.762	12.769	11.296	17342.371	17330.239
Number 7 Cardiac angiography	6.123	8.940	9.965	3336.666	3328.433
Number 8 Angiography	8.929	13.580	13.626	66651.142	66638.937
